# Neglected case of a huge leiomyoma in an elderly postmenopausal woman: a case report

**DOI:** 10.1186/s13256-022-03705-z

**Published:** 2022-12-29

**Authors:** Priyanka Garg, Romi Bansal

**Affiliations:** 1 Present Address: Department of Obstetrics and Gynaecology, All India Institute of Medical Sciences, Bathinda, Punjab 151001 India; 2 Department of Obstetrics and Gynaecology, Adesh Institute of Medical Sciences and Research, Bathinda, 151001 India

**Keywords:** Leiomyoma, Fibroid, Postmenopausal, Leiomyosarcoma

## Abstract

**Background:**

Fibroids are benign tumors of the female reproductive tract originating from the myometrial smooth muscle cells. They are a frequent occurrence in women of childbearing age but their incidence is rare after menopause. In addition, there is a remote possibility of malignant transformation to leiomyosarcoma in less than 1% of cases.

**Case presentation:**

We hereby report a case of large fibroid in a postmenopausal Indian female with rapid growth, raising the suspicion of malignant transformation into leiomyosarcoma. Total abdominal hysterectomy with bilateral salpingo-oophorectomy was performed. Histopathology report confirmed it to be benign leiomyoma without any evidence of neoplasia.

**Conclusion:**

Sudden enlargement of leiomyoma in postmenopausal women should not be ignored due to possible malignant transformation and is to be dealt immediately with hysterectomy followed by histopathology.

## Introduction

Leiomyoma is one of the most frequent benign tumors in the reproductive age group, with an estimated prevalence of 40–50% in women older than 35 years [1, 2]. However, the reported incidence in the postmenopausal age group is as low as 1–2% in the 60–80 year age group [3]. This is attributed to the estrogen-dependent nature of tumor growth, which becomes clinically insignificant after menopause due to the permanent loss of ovarian function. Therefore, a rapid growth noted in the postmenopausal period should point toward a provisional diagnosis of leiomyosarcoma until proven otherwise. Elective hysterectomy followed by histopathology is the definitive management in such cases. We report one such case in an elderly postmenopausal woman who presented to us with a massive abdomino-pelvic mass with sudden enlargement, raising the suspicion of malignancy.

## Case presentation

We present the case of a 74-year-old postmenopausal Indian woman, with four previous normal vaginal deliveries who presented to us with complaints of progressive abdominal lump and feeling of heaviness in the abdomen for the last 7 years. However, the patient continued to ignore these symptoms and did not seek any treatment. Initially, the increase in tumor size was gradual until the last year, when the growth increased rapidly. She also had a complaint of generalized pain in the abdomen for 20 days. There were no associated symptoms of weight loss, gastrointestinal disturbance, breathing difficulties, or abnormal vaginal bleeding. She was postmenopausal for 25 years without any history of postmenopausal bleeding. There was no history of hormone therapy, nor breast, ovarian, or endometrial cancer in the family.

On general physical examination, she was overweight with a body mass index (BMI) of 27 kg/m^2^. Patient vital signs were stable. Abdomen examination confirmed large distended abdomen due to an abdomino-pelvic mass corresponding to 36 weeks of uterus reaching up to xiphisternum and arising from the pelvis, but its lower border could not be palpated. It was dull on percussion, had a smooth surface, was firm in consistency, non-tender, and slightly mobile in the transverse plane. There was no shifting dullness that negated the presence of ascites. Gynecological examination revealed an abdomino-pelvic mass corresponding to 36 weeks of pregnant uterus in size with normal external vulva and cervix. The uterus could not be appreciated separately. Clinical examination was followed by imaging investigations. Ultrasound depicted a well-defined cystic, solid multi-septated mass in the lower abdomen measuring 20.2 × 19.2 cm. It was septated with solid mural modules. Bilateral ovaries and uterus could not be visualized separately. The mass had good vascularity, raising the possibility of ovarian origin and neoplasia. On further evaluation, magnetic resonance imaging (MRI) revealed a large well-defined globular mass measuring 24 × 23.9 × 18 cm originating from the right lateral wall of the uterus, and was suggestive of a sizeable degenerating leiomyoma of the uterus. A provisional diagnosis of uterine leiomyosarcoma was made.

**Fig. 1:**
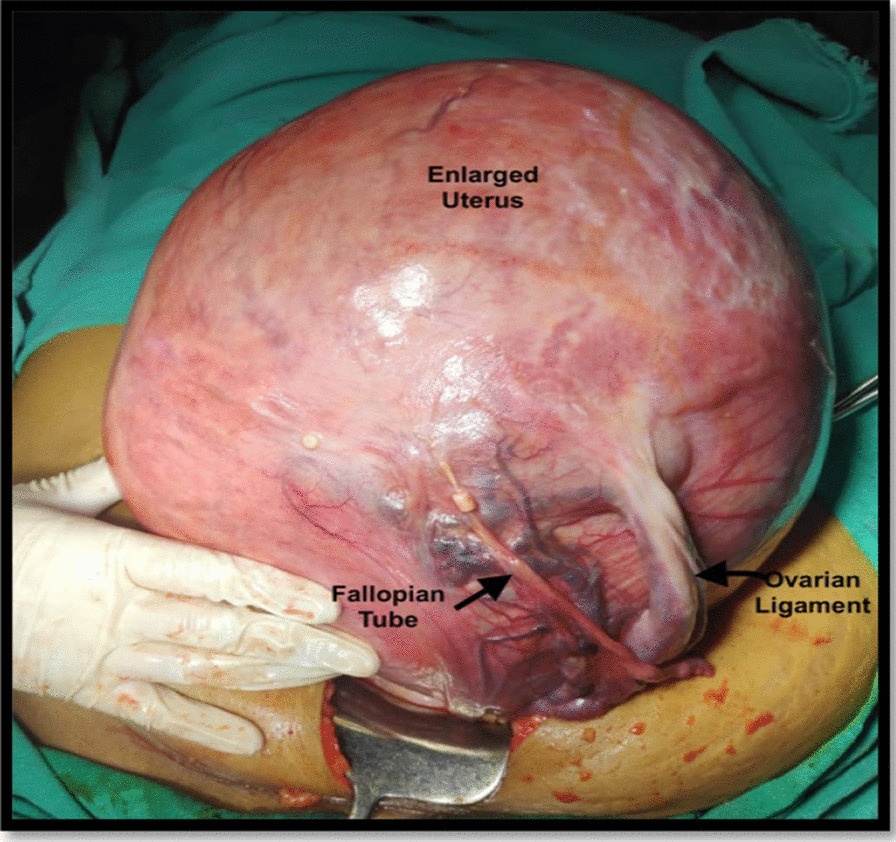
Intraoperative appearance of enlarged uterus with overstretched left adnexa

**Fig. 2:**
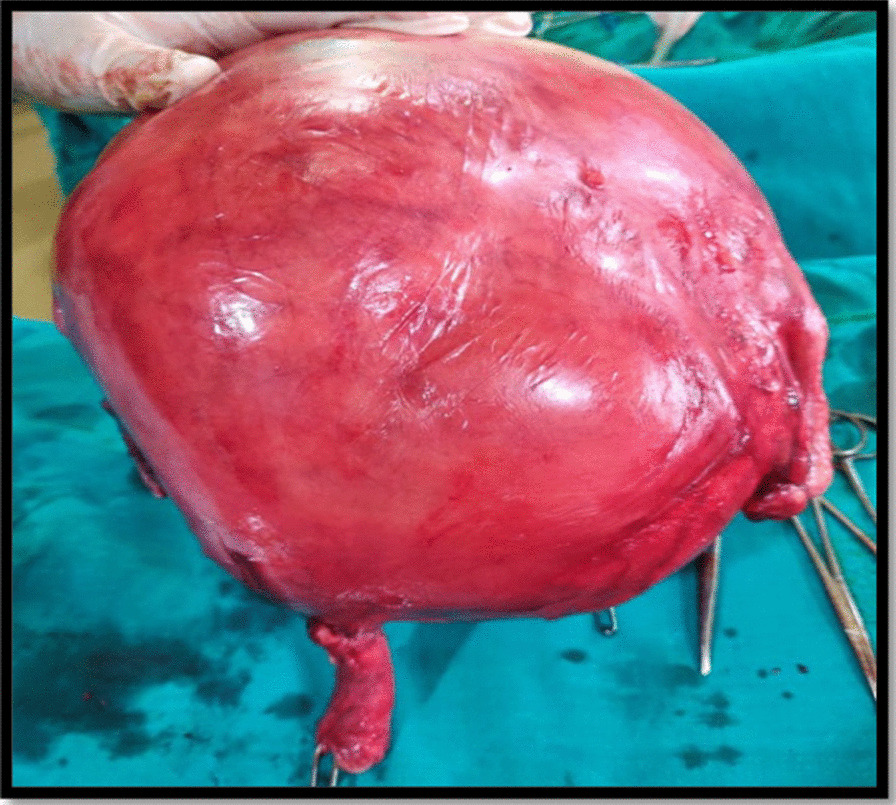
Postoperative specimen showing the uterus with large myoma

**Fig. 3:**
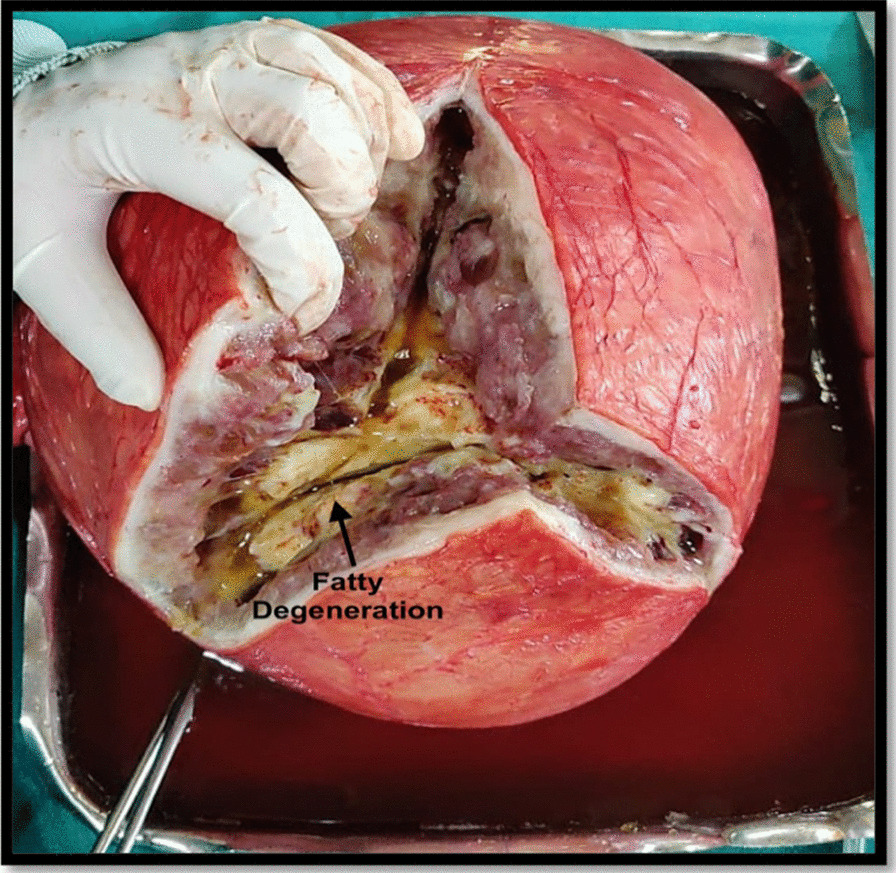
Cut section of specimen showing fatty degeneration

Pre-operatively, the hematological and biochemical investigations were within normal range, including serum CA 125 (25.1 U/ml). After obtaining informed written consent and arranging adequate blood products, patient was undertaken for exploratory laparotomy. The abdomen was opened by a vertical midline incision. Peroperatively, a large intramural fibroid originating from the uterus filling whole of the abdomen was seen. Peritoneal washings were collected and a total abdominal hysterectomy (TAH) with bilateral salpingo-oophorectomy (BSO) was performed (Fig. 1). There was excessive blood loss during the surgery (approximately 3 L). She was transfused three units of packed cells and three units of fresh frozen plasma. The postoperative period was uneventful with satisfactory recovery. The gross histopathological examination showed a 25 × 22 × 16 cm intramural leiomyoma with normal bilateral tubes and ovaries (Fig. 2). The specimen weighed around 7 kg, and the cut section depicted fibro-fatty degeneration (Fig. 3). No malignant cells were seen in peritoneal washings. There was no evidence of sarcomatous changes on histopathology. The final diagnosis was benign intramural uterine leiomyoma with cystic and fatty degeneration. On follow-up visits at 6 weeks, 3 months, and 6 months, the patient was asymptomatic and doing well.

## Discussion

Uterine leiomyomas, commonly called fibroids, are benign smooth muscle tumors of monoclonal origin containing varying amounts of connective tissue [4, 5]. They are common in the reproductive age group and are extremely rare after menopause. Although rare, anecdotal cases of huge fibroids in postmenopausal women have been reported in the literature previously [6, 7]. Their occurrence after menopause could be due to stimulation of the growth by estrone, insulin-like growth factor, or epidermal growth factor [8]. In postmenopausal women with obesity, peripheral aromatization of adrenal-derived androstenedione into estrone might be responsible for the increase in size. As the fibroids enlarge, they outgrow their blood supply or cause mechanical compression of feeder arteries and undergo degenerative changes [9]. Hyaline degeneration (63%) is the most common, followed by myxomatous (13%), calcareous (8%), mucoid (6%), cystic (4%), carneous (3%), and fatty changes (3%). The exact pathogenesis for degeneration of fibroids in postmenopausal women remains unclear, however, increased production of growth factors (epidermal or insulin like) from the fibroid might explain this condition [9]. Rarely, it may undergo malignant degeneration to become leiomyosarcoma in less than 1% of cases [7]. Sadly, the clinical profile of benign leiomyoma and uterine leiomyosarcoma is identical. The definitive diagnosis of a uterine sarcoma can only be made histopathologically after removing the tumor via myomectomy or hysterectomy [6]. Ultrasound is the first line investigation for diagnosing these fibroids, especially in resource-constrained health centers, as it is the least invasive and most economical. MRI must be considered in cases with a large fibroid or a rapidly growing fibroid in a non-acute setting [1].

The patient may be asymptomatic or present with varied symptomatology, such as pelvic pain, irregular vaginal bleeding, acute abdomen, lump in the abdomen, pressure-related symptoms, or rapid increase in size, which was seen in our case. Ghaffar reported a case of a 56-year-old postmenopausal woman who presented with polycythaemia associated with rapid enlargement of the uterus due to a huge fibroid weighing 5.05 kg [3]. Osegi *et al*. reported a case of 58-year-old menopausal female with huge fibroid measuring 22 × 16 × 25 cm deriving its blood supply from the omental vessels. They performed total abdominal hysterectomy with bilateral salpingo-oophorectomy and partial omentectomy. Histopathology revealed it to be benign leiomyoma with cystic changes [4]. Seet *et al*. also reported a 55-year-old menopausal woman with a large degenerating fibroid [1]. Shrestha *et al*. published a case of a menopausal woman who presented with acute abdomen due to fibroid degeneration [9]. Another case of a calcified fibroid was reported in a postmenopausal woman, managed with hysterectomy [8]. Myomectomy or hysterectomy must be planned depending on the patient’s age, type and size of the fibroid, severity of symptoms, desire to conceive in the future, suspicion of malignancy, and proximity to menopause [6]. Since our patient was an elderly postmenopausal woman with rapidly enlarging fibroid raising the suspicion of malignancy, TAH with BSO was the treatment of choice. Histopathology remains the gold standard to rule out the possibility of sarcomatous changes.

## Conclusion

Uterine leiomyomas are a rare entity in the postmenopausal group. The possibility of leiomyosarcoma, although rare, should be borne in mind when such patients present with sudden enlargement of fibroid. The initial clinical presentation is usually misleading, and correct diagnosis can only be made by histopathology. Definitive management in the form of hysterectomy should be offered to such patients to prevent further morbidity and mortality.

## Data Availability

Not applicable.
